# Molecular preservation by extraction and fixation, mPREF: a method for small molecule biomarker analysis and histology on exactly the same tissue

**DOI:** 10.1186/1472-6890-11-14

**Published:** 2011-12-21

**Authors:** Jeffrey R Shuster, Raymond S Lance, Dean A Troyer

**Affiliations:** 1 Current address, The Biomarker Factory, 4307 Emperor Blvd, Ste 200, Durham, NC, USA; 2 Dept of Urology, Eastern Virginia Medical School, Norfolk, VA, USA; 3 Depts. Of Pathology and Microbiology and Molecular Cell Biology, Eastern Virginia Medical School, Norfolk, VA, USA

## Abstract

**Background:**

Histopathology is the standard method for cancer diagnosis and grading to assess aggressiveness in clinical biopsies. Molecular biomarkers have also been described that are associated with cancer aggressiveness, however, the portion of tissue analyzed is often processed in a manner that is destructive to the tissue. We present here a new method for performing analysis of small molecule biomarkers and histology in exactly the same biopsy tissue.

**Methods:**

Prostate needle biopsies were taken from surgical prostatectomy specimens and first fixed, each in a separate vial, in 2.5 ml of 80% methanol:water. The biopsies were fixed for 24 hrs at room temperature and then removed and post-processed using a non-formalin-based fixative (UMFIX), embedded, and analyzed by hematoxylin and eosin (H&E) and by immunohistochemical (IHC) staining. The retained alcohol pre-fixative was analyzed for small molecule biomarkers by mass spectrometry.

**Results:**

H&E analysis was successful following the pre-fixation in 80% methanol. The presence or absence of tumor could be readily determined for all 96 biopsies analyzed. A subset of biopsy sections was analyzed by IHC, and cancerous and non-cancerous regions could be readily visualized by PIN4 staining. To demonstrate the suitability for analysis of small molecule biomarkers, 28 of the alcohol extracts were analyzed using a mass spectrometry-based metabolomics platform. All extracts tested yielded successful metabolite profiles. 260 named biochemical compounds were detected in the alcohol extracts. A comparison of the relative levels of compounds in cancer containing *vs*. non-cancer containing biopsies showed differences for 83 of the compounds. A comparison of the results with prior published reports showed good agreement between the current method and prior reported biomarker discovery methods that involve tissue destructive methods.

**Conclusions:**

The Molecular Preservation by Extraction and Fixation (mPREF) method allows for the analysis of small molecule biomarkers from exactly the same tissue that is processed for histopathology.

## Background

Histopathology is the standard method for cancer diagnosis and grading to assess aggressiveness in clinical biopsies. Molecular biomarkers have also been described that are associated with cancer aggressiveness. A long-standing problem is that while intact tissue is required for microscopic examination for histology, biomarker detection often requires tissue disruption [1] meaning that the histopathology and molecular analysis are not performed on the same exact tissue. Immunohistochemistry (IHC) and fluorescence in-situ hybridization (FISH) were introduced to detect protein and DNA biomarkers in intact tissues, and although these were major technological innovations, IHC in particular is not quantitative and is affected by many variables [2-4]. These methods applied to intact tissue sections are also very difficult to multiplex so that only one or a few biomarker targets can be measured. Because IHC and FISH are interpreted by microscopic examination, results can also vary between observers [5]. Therefore, analysis of biomarkers in intact tissue remains a choke-point for translational medicine. This is particularly evident when limited amounts of tissue are available, as is often the case when small biopsies are obtained by image guided methods in the outpatient setting. Submitting one such core for histopathology and another separate core for quantitative molecular methods is not desirable as it is difficult to be certain that both portions of tissue contained disease.

**Figure 1:**
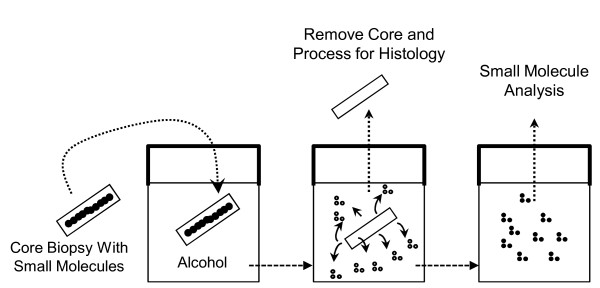
**Molecular Preservation by Extraction and Fixation (mPREF) method**. The mPREF work flow showing, left to right, immersion of tissue biopsy (with small molecules) in alcohol and subsequent removal of the tissue for processing for histology. The remaining alcohol containing the extracted small molecules is capped and stored for subsequent analysis.

This work describes a method that allows for combining histopathology and small molecule biomarker analysis from the same tissue specimen using a fixative solution which historically is only discarded. The method, Molecular Preservation by Extraction and Fixation (mPREF), substitutes alcohol for formalin as a tissue fixative, leading to extraction of small molecules into the alcohol from the tissue (Figure 1). Formalin has been used for tissue fixation in pathology for over 100 years. Form alin's fixative features were accidentally discovered by Ferdinand Blum during experimental studies as a potential disinfectant at the end of the 19^th ^century [6]. The toxic properties of formalin are also well described including carcinogenicity, and acute and chronic exposure related illness [7]. Additionally, it is well known that formaldehyde is largely destructive to small molecules of increasing value to understanding disease states. Using an alcohol extraction, small molecules can be assayed in the biopsy fixative solution while the exact same tissue is processed for histology. Metabolomics is a method by which low molecular weight (<2 kD) biochemical compounds (*e.g*. metabolites) are extracted, detected, and measured. We describe a simple method for combining histology and metabolomics to characterize alcohol extractable small molecules from tissues. The use of alcohol as a tissue fixative is not new, and the accumulated literature suggests that macromolecules (DNA, RNA, and proteins) remain intact in tissue thus processed [8-10]. Therefore, IHC and FISH can be performed on alcohol extracted tissue, and the histopathologist and molecular pathologist can continue to perform the same immunohistochemical and molecular assays now performed on sections of paraffin embedded tissue.

An example for the use of the mPREF methodology is presented for prostate cancer biopsy tissue. Prostate cancer is a significant health concern with an estimated over 200,000 new cases each year in the United States, and although screening for early detection is routinely performed, over 30,000 men die each year from the disease [11]. Prostate cancer is one of the cancers for which screening in asymptomatic populations is currently recommended, and as such, a number of early stage cancers are detected [12-14]. It is well recognized that prostate cancer is a heterogeneous disease [15] and even employing all diagnostic modalities available today, it remains difficult to determine with surety which tumors are indolent, and which are aggressive and have the potential to metastasize. As a result, clinical practice seeks to balance the relative risks of treatments with those of expectant management programs [16,17]. Molecular biomarkers are currently being investigated to help better differentiate more indolent from more aggressive disease. These include the identification of biomarkers based on nucleic acids [18-24], proteins [25,26], metabolites [27-33], advanced histomorphology [25,26], and circulating tumor cells [34-38].

## Results

### Histology and Immunohistochemistry

**Figure 2:**
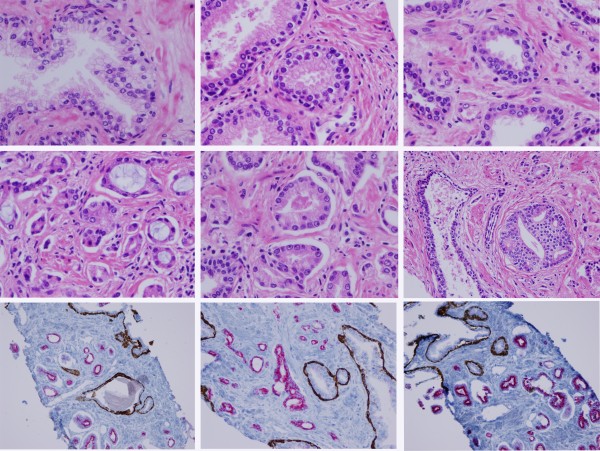
**Composite panel showing H&E and PIN4 IHC of biopsies processed with mPREF**. Histology of prostate biopsies processed using the mPREF method. The figure compares histopathology of benign glands (top row) to malignant glands (middle row) stained with H&E. 4 micron sections were prepared. Each frame is from a separate subject. The bottom row shows IHC staining for PIN-4 cocktail wherein brown basal staining is present in benign prostatic glands and absent in tumor glands. In reciprocal fashion, racemase staining, indicated by red pigment, is present in malignant glands and absent from normal glands. Each frame is from a different subject.

H&E analysis was successful following the pre-fixation in 80% methanol. The presence or absence of tumor could be readily determined for all 96 biopsies analyzed. A montage showing different fields of representative H&E stained histological sections is shown in Figure 2. Previous studies comparing alcohol fixed tissues to formalin fixed tissues focused on nuclear and cellular morphology, architecture, and staining characteristics [10]. We find that each of these features is acceptable in prostate tissues fixed in aqueous alcohol and the alcohol fixation does not interfere with the IHC using the PIN4 cocktail stain (Figure 2). The comparison is qualitative, and there are differences in appearance of alcohol versus formalin fixed tissues including finer nuclear chromatin detail in alcohol. The differences are subtle, however, in most cases, we are confident that trained pathologists can both discern the difference between formalin fixed and alcohol fixed tissues. Further, we are also confident that alcohol fixed tissues can be used for histological diagnosis. We routinely use aqueous alcohol for prostate, bladder, and kidney tissues processed in our clinical facility (Sentara Norfolk General Hospital, Norfolk, Virginia).

### Analysis of metabolites in biopsy extracts

To demonstrate the suitability of mPREF for the analysis of small molecule biomarkers, 28 of the alcohol extracts were analyzed using a robust mass spectrometry-based metabolomics platform. 260 named biochemical compounds were detected in the alcohol extracts. A comparison of the relative levels (by ion counts) of compounds in cancer containing *vs*. non-cancer containing biopsies showed changed levels of 83 of the compounds (matched pairs t-test, p < .05) with 82 of the 83 compounds showing an increased level in extracts from the cancer containing biopsies (Table 1). Eighteen of the 20 common amino acids and a number of long chain fatty acids and phospholipids were increased, possibly indicative of a higher metabolic state in the cancer containing biopsy tissues as compared with non-cancer containing biopsy tissues.

**Table 1:** 

Biochemical Group	Biochemical Pathway	Compound	Fold change Cancer/No-cancer	p-value	q-value
Amino acid	Alanine, aspartate metabolism	alanine	1.6	0.013	0.049
		aspartate	2.2	0.013	0.049
		N-acetylaspartate	6.1	0.020	0.049
	
	Butanoate metabolism	2-aminobutyrate	1.8	0.014	0.049
	
	Cysteine, methionine metabolism	cysteine	4.6	0.049	0.061
		
		cystine	2.9	0.024	0.049
		
		methionine	3.6	0.012	0.049
		
		S-adenosylhomocysteine	1.6	0.004	0.049
	
	Glutamate metabolism	glutamate	2.4	0.019	0.049
		
		N-acetyl-aspartyl-glutamate	1.9	0.014	0.049
	
	Glutathione metabolism	5-oxoproline	1.9	0.017	0.049
		
		cysteine-glutathione disulfide	3.4	0.043	0.060
	
	Glycine, serine, threonine metabolism	betaine	2.3	0.022	0.049
		
		glycine	2.6	0.020	0.049
		
		serine	2.1	0.009	0.049
		
		threonine	1.4	0.003	0.049
	
	Histidine metabolism	histidine	1.5	0.001	0.043
	
	Lysine metabolism	2-aminoadipate	2.1	0.021	0.049
		
		lysine	2.3	0.027	0.052
	
	Phenylalanine, tyrosine metabolism	phenylalanine	1.8	0.011	0.049
		
		tyrosine	1.9	0.014	0.049
	
	Tryptophan metabolism	tryptophan	1.9	0.010	0.049
	
	Urea cycle; arginine, proline metabolism	arginine	1.2	0.033	0.056
		
		ornithine	2.7	0.019	0.049
		
		proline	1.6	0.007	0.049
		
		trans-4-hydroxyproline	2.1	0.036	0.056
	
	Valine, leucine, isoleucine metabolism	isoleucine	1.8	0.016	0.049
		
		leucine	1.8	0.010	0.049
		
		valine	1.6	0.016	0.049

Carbohydrate	Aminosugars	N-acetylglucosamine	5.9	0.028	0.052
	
	Glycolysis	glucose 1-phosphate	0.7	0.033	0.056
	
	Pentose metabolism	ribose	2.7	0.018	0.049

Cofactors and vitamins	Pantothenate	pantothenate	2.3	0.008	0.049
	
	Tocopherol	alpha-tocopherol	2.2	0.041	0.058

Energy	Krebs cycle	fumarate	2.1	0.007	0.049
		
		malate	2.0	0.006	0.049
		
		succinate	1.6	0.009	0.049
		
		succinylcarnitine	2.2	0.041	0.058

Lipid	Carnitine metabolism	3-dehydrocarnitine	1.9	0.023	0.049
		
		acetylcarnitine	1.6	0.015	0.049
		
		carnitine	1.7	0.033	0.056
		
		deoxycarnitine	1.6	0.010	0.049
	
	Essential fatty acid	docosahexaenoate	3.2	0.046	0.060
		
		docosapentaenoate	4.2	0.018	0.049
	
	Fatty acid metabolism	butyrylcarnitine	1.9	0.012	0.049
		
		propionylcarnitine	2.0	0.036	0.056
		
		4-hydroxybutyrate	2.1	0.023	0.049
	
	Glycerolipid metabolism	choline	1.7	0.004	0.049
		
		ethanolamine	3.4	0.039	0.056
		
		glycerol	1.9	0.047	0.060
		
		glycerophosphorylcholine	1.8	0.022	0.049
	
	Long chain fatty acid	10-nonadecenoate	2.8	0.036	0.056
		
		dihomo-linoleate	4.8	0.038	0.056
		
		eicosenoate	3.0	0.013	0.049
		
		myristoleate	1.7	0.045	0.060
		
		oleate	2.1	0.038	0.056
	
	Lysolipid	1-arachidonoylglycerophosphoethanolamine	1.9	0.007	0.049
		
		1-arachidonoylglycerophosphoinositol	2.2	0.006	0.049
		
		1-linoleoylglycerophosphoethanolamine	2.6	0.020	0.049
		
		1-oleoylglycerophosphoethanolamine	2.4	0.007	0.049
		
		1-oleoylglycerophosphoinositol	3.7	0.035	0.056
		
		1-oleoylglycerophosphoserine	2.6	0.022	0.049
		
		1-palmitoylglycerophosphoinositol	3.3	0.047	0.060
		
		1-stearoylglycerophosphoethanolamine	2.2	0.019	0.049
		
		1-stearoylglycerophosphoinositol	4.0	0.044	0.060
		
		2-oleoylglycerophosphoethanolamine	2.4	0.004	0.049
		
		2-palmitoylglycerophosphoethanolamine	2.1	0.027	0.052
	
	Medium chain fatty acid	caprylate	1.2	0.046	0.060

Nucleotide	Purine and pyrimidine	methylphosphate	2.2	0.018	0.049
	
	Purine metabolism, hypoxanthine/inosine	hypoxanthine	2.2	0.004	0.049
		
		inosine	1.6	0.026	0.052
		
		xanthine	5.2	0.030	0.055
		
		xanthosine	2.0	0.014	0.055
	
	Purine metabolism	adenine	1.8	0.038	0.056
		
		guanosine	1.6	0.035	0.056
	
	Pyrimidine metabolism	cytidine	1.9	0.034	0.056
		
		pseudouridine	1.3	0.024	0.049
		
		uracil	4.0	0.003	0.049
		
		uridine	1.9	0.008	0.049

Peptide	gammaglutamyl	gamma-glutamylglutamate	2.3	0.032	0.056
		
		gamma-glutamylglutamine	3.3	0.033	0.056

Other	Benzoate metabolism	benzoate	1.4	0.045	0.060
	
	Other	glycerol 2-phosphate	2.1	0.028	0.052

**Figure 3:**
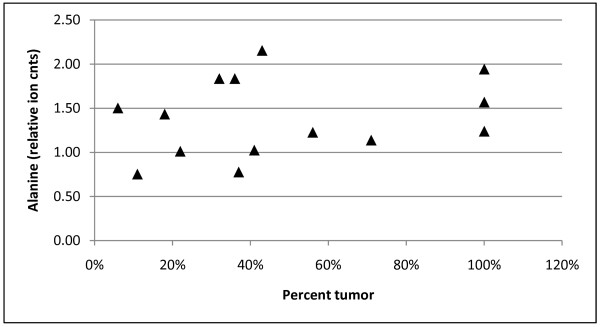
**Alanine levels as a function of percent tumor in biopsies**. The percent tumor in the cancer containing biopsies was determined by dividing the length of tumor by length of the core. The scaled alanine ion counts were determined as described in Materials and Methods.

It was not feasible to obtain biopsies with the same amount of tumor present in each of the needle biopsies. The average percent of tumor in the biopsies for pathology T3 tumors was higher than that of the T2 tumors (60% for T3 biopsies, and 36% for the T2 biopsies). To determine the relationship of compound level as a function of the percent tumor in the biopsy, the levels for one of the amino acids, alanine, that has been previously reported to increase in prostate cancer [28] was plotted as a function of the percent tumor measured in the biopsy. Figure 3 shows that there is not a strong relationship between percent of tumor in the biopsy and the level of alanine. Similar results were observed for other amino acids (not shown).

The metabolomics method used in this study is semi-quantitative and reports relative levels for each of the compounds as ion counts (absolute levels of metabolites are not determined using this method). The highest fold changes observed for the 83 compounds were approximately 4- to 6-fold with the following compounds having over 4-fold increases in cancer biopsy extracts: cysteine, dihomo-linoleate, docosapentaenoate, N-acetylaspartate, N-acetylglucosamine, uracil, xanthine, and 1-stearoylglycerophosphoinositol (Table 1).

The purpose of this study was primarily to demonstrate that histopathology and small molecule biomarker marker studies could be performed on the same exact tissue specimen. To also determine whether the tissue non-destructive mPREF method results were consistent with prior reported studies that used tissue grinding methods, a subset of metabolites was examined based on published reports of small molecule candidate biomarkers that change with the degree of aggressiveness of prostate cancer. Although the number of biopsy extracts analyzed by mass spectrometry were limited in number, the results were consistent with prior published data with similar numbers of prostatectomy tissues as described below.

**Figure 4:**
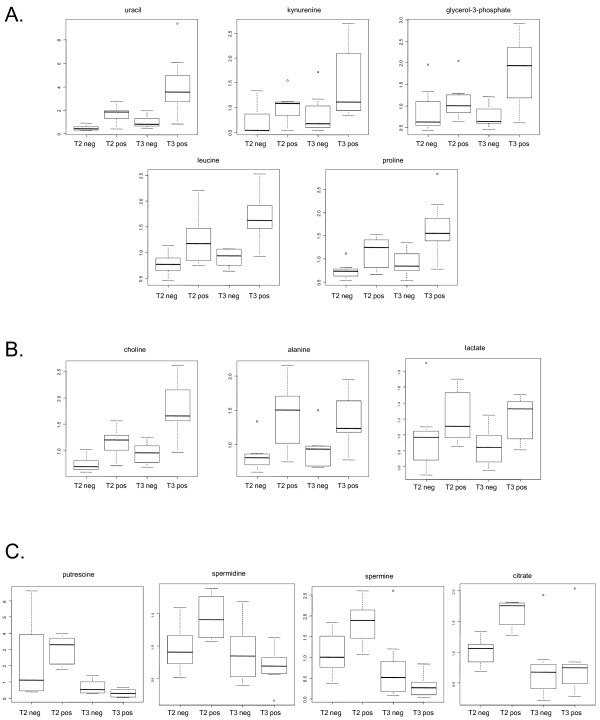
**Relative levels of metabolites from alcohol extracts of biopsies using the mPREF method categorized by prostatectomy pathology class**. Box and whisker plot distributions; box lower quartile to the upper quartile; whiskers, minimum and maximal values without outliers; line, median value. x-axis categories: T2, pathology stage T2, organ-confined disease; T3, pathology stage T3, non-organ-confined disease; neg, biopsy did not contain tumor; pos, tumor present in biopsy. y-axis: relative amount based on mass spectrometry ion counts.

Sreekumar *et al. *[27], examining 16 benign, 12 localized prostate cancer, and 14 metastatic prostate cancer tissues by metabolomics using a tissue grinding method reported that, "A subset of six metabolites including sarcosine, uracil, kynurenine, glycerol-3-phosphate, leucine and proline were significantly increased on disease progression from benign to PCA [organ localized prostate cancer] to metastatic prostate cancer." Therefore, the relative levels for 5 of 6 of these compounds were examined (sarcosine was below the detection limit in this study) comparing the less aggressive T2 organ-confined disease and the more aggressive T3 non-organ-confined disease. All five compounds were observed to be higher in biopsy extracts from cancer containing biopsies than non-cancer containing biopsies, and the levels for all 5 compounds were higher in extracts from pathology T3 prostates than from T2 prostates (Figure 4 panel A). Therefore, the results obtained using the mPREF method were consistent with the prior published data for these candidate biomarkers.

Additional small molecule compounds have been reported to differ in benign *vs*. cancer containing prostate tissues using non-mass spectrometry-based methods of analysis including magnetic resonance spectroscopy imaging, MRSI. These include increases in cholines, lactate, and alanine, and decreases in polyamines and citrate [28,30,33,39-42]. The levels of these compounds were also examined in the biopsy extracts from the mPREF method. The results (Figure 4 panel B) show that choline could differentiate cancer from non-cancer, and T2 from T3 disease, whereas alanine and lactate appeared to be able to differentiate cancer from non-cancer, but did not show a marked increase from T2 to T3 disease. These results also demonstrate that the mPREF method yields results consistent with previously published data. The polyamines, putrescine, spermidine, and spermine, and citrate had more complex patterns (Figure 4 panel C). These compounds appeared to increase in level in T2 prostate biopsies as compared with non-cancer biopsies, but decreased in relative amounts in T3 prostate biopsies.

## Discussion

We report here a new method for the extraction of molecular biomarkers from human tissue that is non-destructive to the tissue and is compatible with downstream workflow including histopathology and immunohistochemical analysis. The method involves the simple immersion of the tissue in 80% methanol (aqueous) which both extracts the small molecules and fixes the tissue. The analysis of the tissue extracts has the potential to provide for identification of molecular biomarkers associated with any biological process in a wide array of different tissue types. The method is compatible with small tissue specimens such as are obtained by needle biopsies and therefore has potential utility for clinical practice.

Histology is the gold standard for classifying disease and assigning prognostic grade, particularly in oncology. The direct examination of the architecture of intact tissue sections by pathologists remains powerful in classifying disease, however the data generated is usually qualitative, or semi-quantitative and the methods for preparing histological sections can be limiting for some downstream analyses. Studies of precision in histology have shown that scoring is generally consistent, however, there is not always full agreement across different laboratories [43-46]. Analysis of molecular biomarkers provides for the possibility of quantitative measurements to augment histology in the characterization of disease. Innovations intended to improve biomarker detection must take into account several factors. Biomarkers originating from tissues have often proven difficult to discover in blood because of the dilutive factor. For example, the most frequently used biomarker for the early detection of prostate cancer, PSA, is not routinely seen in proteomics discovery studies in blood from prostate cancer patients because of its low levels. To increase the probability of biomarker discovery, the closer in proximity one can get to the diseased tissue, the more concentrated will be disease specific proteins, peptides, nucleic acids, and metabolites. The mPREF method described in this study addresses many of the issues for the discovery of biomarkers and their translation to clinical practice. It is compatible with the long established diagnostic and prognostic histology-based methods, and IHC and FISH can still be performed on the paraffin embedded tissue. The small molecule biomarkers appear to reflect meaningful biological differences amongst tumors, and assays for these classes of molecules can be developed that are cost effective, high throughput, and highly accurate.

The use of alcohols as tissue fixatives is not new to histology and has been recently described as an alternative to formaldehyde-based fixatives [8-10]. We have demonstrated that alcohol fixation of prostate biopsies using the mPREF method produces tissue histology slides that can be readily read for the presence or absence of cancer (Figure 2). In addition, IHC with the PIN4 cocktail stain demonstrated good resolution of basal cells and basal keratin in normal prostate glands. In prostatic adenocarcinoma, basal cells and basal keratin were absent while racemase was detected in the cytoplasm. Other IHC stains will need to be tested empirically to determine their compatibility with the mPREF method. It is recognized that any change of fixation procedure has the potential to change the appearance of the tissues and that a larger number of tissues will need to be examined before general utility can be validated. The drivers for any change in procedures are related to the strength of the utility of any new biomarkers for the classification of disease. It was not the purpose of this study to extensively validate the histology and staining characteristics of alcohol *vs*. formalin as a fixative for prostate biopsies. We recognize that most pathologists are trained using formalin fixed tissues, however, pathologists must also read cytology, hematopathology smears and cryosections, which are frequently fixed in aqueous alcohol. We anticipate the formation of a study group of interested pathologists to share slides and more formally evaluate this method's acceptability for diagnosis and grading of disease.

The primary goal of this work was to describe a new method for the analysis of molecular biomarkers from human tissue that allows for histopathology to be performed in the same exact tissue. In addition, although the numbers of samples analyzed by mass spectrometry was limited in number, the mPREF method produced data that is consistent with published reports for candidate biomarkers for prostate cancer. Metabolite candidate biomarkers previously identified using a tissue grinding method and reported to increase in the progression of prostate cancer [27] also showed increased levels with the mPREF method when comparing cancer *vs*. non-cancer. The levels of these candidate biomarkers were also increased in more aggressive non-organ-confined (T3) disease, *vs*. the less aggressive organ-confined (T2) disease (Figure 4A). Other compounds also previously reported to be increased in prostate cancer [28,30,39-41], were also observed to be increased in cancer containing biopsies using the mPREF method (Figure 4B). The relatively small numbers of prostate biopsy extracts analyzed for metabolite profiles in this study precluded an in depth statistical analysis, however, further experiments are planned to investigate these results in more detail.

In this study, 82 compounds were observed to increase in cancer *vs*. non-cancer biopsy extracts. Eighteen of the 20 common amino acids and a number of long chain fatty acids and phospholipids were increased. Analysis of the amino acids showed that the increase in the levels of these compounds is not directly related to the percent of tumor in the biopsy (Figure 3). It is possible that the metabolite levels have a degree of a zone effect showing an increase in amount in areas close to histopathologically confirmed tumor. The compounds with the highest fold increases observed (4-6 fold) were cysteine, dihomo-linoleate, docosapentaenoate, N-acetylaspartate, N-acetylglucosamine, uracil, xanthine, and 1-stearoyl-glycerophosphoinositol. It is difficult to know which of the compounds are increased as a result of a general higher metabolic rate in more rapidly dividing cells, and which, if any, compounds represent changes in metabolism specifically associated with the oncogenic process. With regard to the potential utility of these types of changes, it has been demonstrated that the expression of cell proliferation-related genes has prognostic value for cancer disease progression [47], and it will be of interest to determine in later experiments which of the metabolites may have similar utilities.

The polyamines have been well studied as biomarkers in oncology [30,41,42]. In cancers of non-prostatic origin, polyamines are increased, yet, as a differentiated function in the prostate, the polyamines decrease in prostate cancer as the tissue becomes de-differentiated. In this study, the polyamines were observed to be the lowest levels from the highest grade cancer (T3) containing biopsies (Figure 4C). This is consistent with polyamines decreasing in prostate cancer, however, the polyamines were higher in pT2 cancer containing biopsies as compared with non-cancer containing biopsies.

With the notable exception of genomic DNA-based analysis, the discovery and performance of molecular biomarkers is highly dependent upon clinical sampling, processing, and storage [48-50]. In this study, we describe the mPREF method for prostate needle biopsy specimens obtained from fresh prostate tissues following prostatectomy. The future translation of this work to clinically relevant studies will be to include biopsies obtained *in vivo *using TRUS guided trans-rectal biopsy procedures. The use of image guided small core needle biopsies is likely to continue to increase while excisional biopsies decrease in part because the latter are more invasive, can cause more scarring and deformation, and require use of the operating room, the most expensive location in a hospital [51]. The use of core needle biopsies for molecular testing such as mutational analysis to identify therapeutic targets provides a new rationale both for initial diagnosis and for repeat core needle biopsies to monitor therapy. Examples include *EGFR *mutation analysis in lung cancer [52] and *HER-2 *in breast cancer [53]. The mPREF method described in this study should be widely applicable to a variety of tissues. The approach described has no inherent limitations for tissue type or disease state. While providing a method for quantitative analysis of small molecules, it is also compatible with histology, mutational analysis, RNA expression, immunohistochemical analysis, and other *in situ *methods.

## Conclusions

This paper describes a simple method, mPREF, for the extraction and analysis of small molecular weight biomarkers from tissue specimens that is non-destructive to tissue and is compatible with downstream histology analysis, such that histopathology and biomarker analysis can be performed on the same exact tissue. The method is compatible with clinical biopsies without undue interruption in clinical practice for pathology.

## Methods

The mPREF method allows for extraction and measurement of low molecular weight biomarkers and for histology to be performed on exactly the same portion of tissue. A summary diagram of the mPREF method is shown in Figure 1.

### Biopsies

Eight post-operative prostates were obtained from consented subjects for inclusion in a biorepository approved by the Institutional Review Board at Eastern Virginia Medical School. The prostates were removed surgically as primary treatment for prostate cancer. The clinical pathology characterizations of the prostates used in this study are shown in Table 2. Needle biopsies were taken from the prostates *ex vivo *with an 18 gauge core needle biopsy gun. The needle biopsies were taken in a manner to follow, as closely as possible, the sampling pattern utilized for an *in vivo *12-core biopsy sampling protocol. The peripheral zone is selectively oversampled, with left and right lateral and medial biopsies obtained from apex, mid-, and base of the prostate. The cores routinely obtained were 0.9-1.2 cm in length and weighed approximately 5-6 mg. A total of 96 prostate biopsies were obtained.

**Table 2:** 

Prostatectomy Reference Number	Pathology Gleason	Pathology Stage	Margin Status	Biopsy Positive Cores Selected for Small Molecule analysis (n)	Biopsy Negative Cores Selected for Small Molecule analysis (n)
21	4+3 = 7	pT2aNxMx	negative	1	2

28	3+4 = 7	pT3aNxMx	negative	1	1

33	3+3 = 6	pT2cNxMx	negative	2	2

34	3+4 = 7	pT2cN0Mx	negative	2	1

41	4+5 = 9	pT3aN0Mx	multifocal	2	2

42	3+4 = 7	pT2cN0Mx	negative	2	2

44	3+4 = 7	pT3aN0Mx	focal	2	2

51	4+3 = 7 (tert 5)	pT3bN0Mx	multifocal	2	2

### Method of Biopsy Tissue pre-Fixation, Fixation, and post-Processing

Each prostate needle biopsy core was placed immediately into a separate 1.8 ml vial (Thermo Scientific #375418) containing 1.0 of 80:20 vol/vol, methanol:water. No buffers were used in this formulation so as not to interfere with later downstream sample concentration and mass spectrometry analysis. The lids were screwed tightly onto the vials to limit evaporation, and the biopsy cores were fixed in the aqueous alcohol solution at room temperature, without shaking, for 24 hr. The following day, cores were transferred to a commercially available non-formaldehyde-based fixative, UMFIX (Sakura Finetek USA, Inc., Torrance, California), until processed. This commercial fixative is an alcohol based fixative. Biopsies were processed on a Sakura Express × 50 automated processor with an approximate run time of 1.5 hrs. Tissue was embedded immediately following processing. Histology sections were prepared using the following technique. Blocks are faced, and placed in a freezer to chill. A smooth block of ice is prepared to receive the chilled blocks. Paper toweling is spread evenly over the ice and saturated with a solution of ammonia water (900 ml water: 30 ml ammonium hydroxide). Blocks are soaked on the saturated toweling for 1 hour. Biopsies are sectioned at 4 microns, two sections per level, with three levels placed on each slide. One slide is stained with hematoxylin and eosin by routine methods, and one is retained for immunohistochemistry.

### Immunohistochemistry

PIN-4 pre-diluted cocktail (P504S, HMW Cytokeratins, and p63; Cat # PPM 225DS) was purchased from Biocare Medical, Concord, CA. The Ventana BenchMark XT automated stainer was used to process the samples.

### Metabolomic analysis

Fourteen cancer containing cores, and 14 patient matched non-cancer containing cores were selected for metabolomic analysis of the alcohol fixatives (see Table 2). For the cancer containing cores, 7 were from pathologic stage T2 (organ confined disease) prostates and 7 from stage T3 (non-organ-confined disease) prostates. Metabolomic analysis of the biopsy alcohol extracts was performed using a commercial services supplier (Metabolon, Durham, NC), and detailed methods, including sample preparation, instrumentation, conditions for mass spectrometry (liquid chromatography/tandem mass spectrometry in positive and negative ion modes, and gas chromatography/mass spectrometry), peak data reduction, and assignment of peaks to known chemical entities by comparison to metabolite library entries of purified standards, have been previously described [54,55], with the modification that the sample extracts were first concentrated to dryness by evaporation of the 80% methanol:water solvent prior to analysis. The data output from this global metabolomics method is relative ion counts; the absolute quantitative amount of metabolites in a sample is not determined using this method. Instrument variability was determined by calculating the median relative standard deviation (RSD) for internal standards that were added to each sample prior to injection into the mass spectrometers. The median instrument variability was 4%. Overall process variability was determined by calculating the median RSD for all endogenous metabolites (non-instrument standards) present in 100% of technical replicates of pooled experimental samples. The median process variability was 10%. Comparisons of relative ion counts were made following log transformation and imputation with minimum observed values for each compound. Each biochemical was re-scaled to have median equal to 1. Matched-pairs t-tests were used to identify biochemicals that differed significantly between groups.

## Competing interests

JRS is currently an employee of Labcorp, owns stock in Teotten Diagnostics and in Metabolon. JRS, RSL, and DAT are co-inventors on Patent Provisional Application No. 61/346,228 "Methods and Reagents for Metabolomics and Histology in a Biological Sample and a Kit For the Same"

## Authors' contributions

JRS, DAT, and RLS, formulated ideas for the mPREF method. JRS performed data analysis for the metabolomics data, and drafted the manuscript. RSL recruited patients, and guided the procurement and sampling of specimens. DAT procured and banked the specimens, participated in drafting the manuscript and supervised the histology and reading of histopathology slides. All authors have read and approved the final manuscript.

## Pre-publication history

The pre-publication history for this paper can be accessed here:

http://www.biomedcentral.com/1472-6890/11/14/prepub
